# Hybrid surgery in treatment of pulmonary sequestration with abdominal aorta feeding vessel: a case report

**DOI:** 10.1186/s13019-018-0733-6

**Published:** 2018-05-18

**Authors:** Haining Zhou, Shoujun Tang, Quanshui Fu, Li Yu, Lunxu Liu

**Affiliations:** 1 Department of Thoracic surgery, West China Hospital, Sichuan University, Chengdu, 610000 China; 2 Department of Thoracic surgery, Suining Central Hospital, Suining, 629000 China; 3 Shehong People’s Hospital, Suining, 629000 China

**Keywords:** Pulmonary sequestration, Hybrid surgery, Video-assisted thoracoscopic surgery [VATS], Embolization

## Abstract

**Background:**

Pulmonary sequestration is a rare congenital pulmonary dysplasia, which requires surgical resection (either via open thoracotomy or video-assisted thoracoscopic surgery [VATS] or via endoluminal occlusion of the abnormal feeding vessel).

**Case presentation:**

We described a 51-year-old female patient with a history of recurrent cough and repeated pneumonia. She was referred to our hospital for further work-up of pulmonary sequestration. We performed a hybrid surgery (i.e., embolization of the aberrant feeding vessel of the sequestration combined with wedge resection of the left lower lobe lesion through VATS). The patient was discharged on the sixth postoperative day in good condition and without complications.

**Conclusions:**

We believe that a hybrid operation is safer, more feasible, and more comprehensive than other treatments.

## Background

Pulmonary sequestration (PS) can cause recurrent infection and haemoptysis and contrast enhanced computer tomography (CT) is frequently used for the imaging diagnosis of PS so far. Moreover, the gold standard for PS diagnosis is digital subtraction angiography (DSA) or to find the abnormal feeding artery during surgery. Currently, embolism, ligation of the abnormal feeding artery and resection of the diseased lung tissue are performed to treat PS [1]. In this case report, we present a patient with PS successfully treated through a hybrid operation.

## Case presentation

**Fig. 1:**
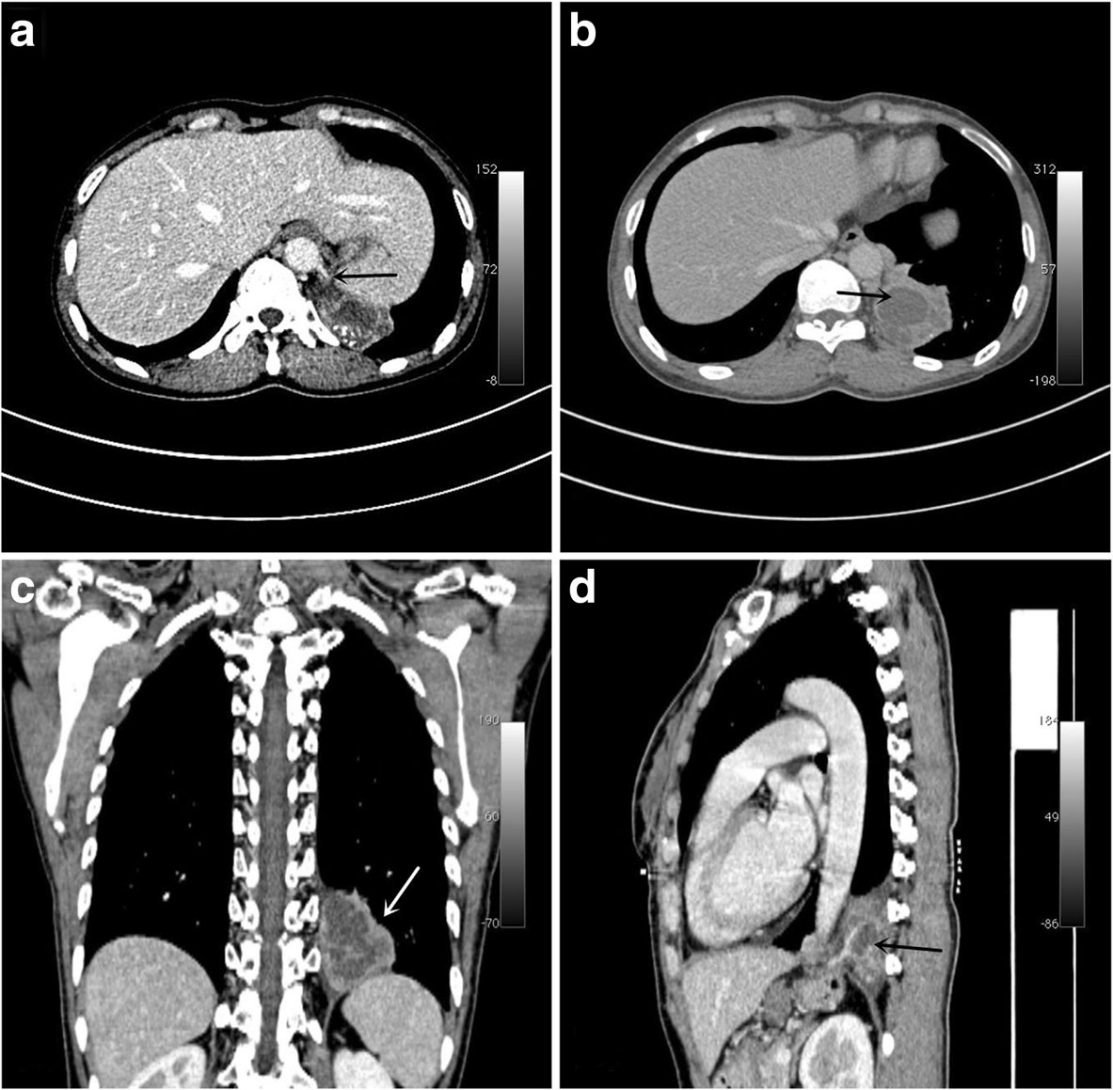
**a** Enhanced CT scan: abnormal blood vessels from the abdominal aorta; **b** mass and abnormal blood vessels in the mediastinum; **c** coronal plane; and **d** sagittal plane

A 51-year-old female patient was admitted to our hospital because of recurrent cough and expectoration for 2 years. Her condition recurred several times intermittently and was exacerbated 7 days before her hospitalization. The patient had no history of smoking, chronic bronchitis or pulmonary tuberculosis. Plain chest CT images showed para-aortic opacities in the left lower lobe. Furthermore, fiberoptic bronchoscopy and abdominal colour Doppler ultrasound showed no significant abnormalities. However, the re-examination of contrast-enhanced chest CT revealed a 45 mm × 41 mm opacity in the left lower lobe and a feeding vessel was found connected to the abdominal aorta. Thus, a tentative diagnosis of PS was made (Fig. 1).

**Fig. 2:**
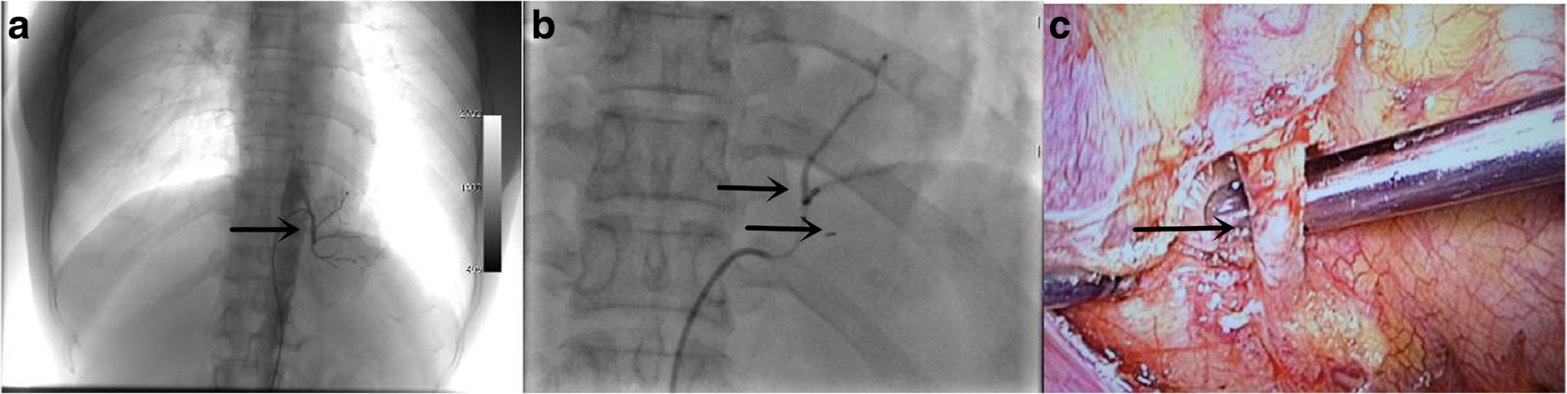
**a** Angiography of the aberrant artery before the interventional embolization; **b** after the interventional embolization; and **c** the aberrant artery clipped by VATS

After completion of preoperative preparation, first the patient underwent an isolated feeding artery embolization under local anaesthesia in the hybrid operating room. A 5F catheter was inserted by Seldinger technique along the right femoral artery. The contrast catheter was selectively inserted into the feeding artery of the left lower lobe PS to perform DSA. Contrast medium was observed in the left cardiophrenic angle and the feeding artery was significantly thickened. Spring rings with different specifications were pushed through the catheter until embolism was satisfactory. Afterward, under general anaesthesia and double-lumen intubation, with the patient in the right decubitus position, a wedge resection of the PS was performed by VATS. A 4-mm aberrant branch from the abdominal aorta was seen to ascend through the diaphragm into the chest to feed the PS. The artery was carefully dissected, ligated with Hem-o-lock avoiding the site of the spring coil to ensure proper closure of the artery, and cut. Finally, a wedge resection of the diseased lung was performed with staplers (Fig. 2). Pathological examination of the resected specimen verified the diagnosis of PS. Postoperative course was uneventful and the patient was discharged on the sixth postoperative day.

## Discussion

The diagnosis of PS mainly depends on the presence of an abnormal feeding artery. The contrast-enhanced CT often shows the feeding artery originating from the aorta [2]. Among the total blood supplied, 70% originates from the thoracic aorta. In the case herein reported, the feeding artery originated from the abdominal aorta. Intralobar PS shares a common visceral pleura with the surrounding lung tissue and has pathological communication with normal bronchi. Thus, repeated infection and haemoptysis are observed during the development of the disease.

PS is mainly treated by resection. In adult patients or children who cannot tolerate surgery, embolization can be used. However, embolization alone with no resection of the PS is unsuitable for patients who have multiple feeding arteries or prolonged and repeated infections before the operation. Presently, most patients undergo either resection or non-surgical methods. Up to now, few cases of endovascular embolization combined with VATS resection of PS have been reported. Avsenik and colleagues reported a case of PS with aneurysmal feeding vessel. Thoracic endovascular aortic repair was performed primarily and, secondarily, a right lower lobectomy was performed. Bhatt and Deutsch described a case of coil embolization of the feeding systemic vessel earlier the day before the planned VATS procedure [3–5]. In this case, a patient with chronic infection experienced pain, discomfort, and other symptoms. Complete removal of the focus of infection in the diseased lung is difficult and can easily induce more infection. A single interventional treatment cannot completely address this problem. Moreover, the feeding artery originates from the abdominal aorta and has systemic pressure. In addition, in the anomalous artery, the muscle fibers are lesser than in the normal arteries and are thus susceptible to tearing, which can lead to massive bleeding or retraction into the peritoneum. A hybrid operation not only can help us clarify the diagnosis and discover the presence and characteristics of the abnormal feeding artery and its branches, but also can facilitate the next step of the surgical procedure. Furthermore, as prolonged and repeated infections could inevitably lead to pleural and diaphragmatic adhesions, a hybrid operation can effectively assist the surgeons in such a way that they can avoid overlooking small branches or causing massive bleeding, thus ensuring the safety of the operation. Compared with a single operation or a single non-surgical procedure, hybrid surgery has broader indications and thus it is more efficient, accurate, and comprehensive. In addition, intraoperative and postoperative complications are relatively few.

## Conclusions

In patients diagnosed with PS who present with a long course of the disease and repeated infections, the lesion is often surrounded by sever adhesions. Moreover, the high-pressured feeding artery from the aorta is highly susceptible to tearing because of inadequate muscle fibers. Thus, for the prevention of catastrophic consequences of an intraoperative accidental injury (such as massive bleeding caused by feeding artery retraction into the peritoneum), we believe that a hybrid operation is safer, more feasible, and more comprehensive than other treatments.

## Abbreviations

CT: Computer tomography
DSA: Digital subtraction angiography
PS: Pulmonary sequestration
VATS: Video-assisted thoracoscopic surgery
